# Endogenous APP-Associated Proteomics in Mouse Brain Defines a Candidate Resource for APP Trafficking and Proteostasis Studies

**DOI:** 10.3390/biomedicines14102293

**Published:** 2026-10-09

**Authors:** Yeting Zeng, Shushan Wei, Keyi Hu, Ruoqi Zhao, Shiwen Yu, Tong Yu, Biqing Huang, Lijie Duan, Xiangteng Zhao

**Affiliations:** 1 Department of Neurology, Fujian Provincial Hospital, Fuzhou University Affiliated Provincial Hospitall, Fuzhou 350001, China; 2 Department of Neurology, Shengli Clinical Medical College of Fujian Medical University, Fuzhou 350001, China; 3 Department of Neurosurgery, Fujian Provincial Hospital, Fuzhou University Affiliated Provincial Hospital, Fuzhou 350001, China; 4 State Key Laboratory of Brain Function and Disorders, Institutes of Brain Science, Fudan University, Shanghai 200433, China; 5 Department of Neurology, Zhongshan Hospital Affiliated to Fudan University, Shanghai 200433, China; 6 Department of Nuclear Medicine, Shengli Clinical Medical College of Fujian Medical University, Fujian Provincial Hospital, Fuzhou University Affiliated Provincial Hospitall, Fuzhou 350001, China; 7 Fujian Research Institute of Nuclear Medicine, Fuzhou 350001, China; 8 Department of Neurology, Jinshan Hospital Affiliated to Fudan University, Shanghai 201500, China

**Keywords:** amyloid precursor protein, endogenous tagging, affinity proteomics, mouse cortex, Gene Ontology, protein candidates

## Abstract

**Background/Objectives:** Affinity proteomics of amyloid precursor protein (APP) is complicated by proteolytic processing, dynamic trafficking, and purification background. We generated a C-terminal 3×FLAG knock-in at the endogenous *App* locus to establish a cortical affinity-proteomic resource and prioritize APP-associated candidates for further study. **Methods:** Eight-week-old male homozygous APP-3×FLAG mice on a C57BL/6J background and wild-type littermates were characterized by biochemical and behavioral assays (n = 6 and n = 7 per genotype, respectively). Cortical material from three APP-3×FLAG males was pooled for anti-FLAG affinity purification and liquid chromatography–tandem mass spectrometry. Protein accessions were filtered by positive Area, unique-peptide support, and control recovery, with Gene Ontology (GO) analyses conducted under alternative backgrounds. Selected candidates underwent targeted co-immunoprecipitation and immunoblotting. **Results:** Baseline APP, amyloid-β, and behavioral measurements showed no statistically significant genotype-associated differences. Five shared high-confidence APP regions showed local Cα RMSD values below 0.3 Å in structural-model comparisons. Across APP-IP and control datasets, 1874 accessions were identified. Evidence filtering retained 990 non-bait candidate accessions representing 967 APP-IP protein groups. Two de novo-only spectra supported recovery of the engineered APP–3×FLAG junction peptide. GO analysis identified 21 significant cellular-component terms under the observed-union background, with none under the evidence-eligible background. Targeted anti-FLAG co-immunoprecipitation and immunoblotting supported ANXA2 and RACK1 co-recovery with APP-3×FLAG. **Conclusions:** The endogenous tagging model, evidence-filtered proteomic dataset, and targeted biochemical findings establish a candidate resource for investigating APP-associated assemblies and prioritizing proteins for studies of trafficking and proteostasis.

## 1. Introduction

Alzheimer’s disease (AD) is a progressive neurodegenerative disorder characterized by amyloid-β (Aβ) deposition, tau pathology, synaptic dysfunction, and neuronal loss [1,2,3,4]. The amyloid precursor protein (APP) remains central to AD biology because Aβ peptides are generated from APP through sequential proteolytic processing by β-secretase and γ-secretase [5,6,7,8,9]. However, APP is not merely a passive precursor of Aβ. It is a type I transmembrane protein with physiological roles in neuronal development, adhesion, synaptic function, and intracellular trafficking, and its processing fate is tightly coupled to its cellular itinerary [10,11,12,13].

APP processing is spatially organized across biosynthetic, secretory, plasma-membrane, endocytic and endolysosomal compartments [10,11,12,13]. These trafficking routes influence whether APP encounters α-, β- or γ-secretase-containing environments, thereby shaping the balance between non-amyloidogenic processing, amyloidogenic processing and APP fragment turnover [10,11,12,13]. Endosomal and lysosomal pathways are particularly relevant to AD pathogenesis. Early endocytic abnormalities have been detected in AD and Down syndrome, and endosomal–lysosomal dysfunction is increasingly viewed as an integral component of AD-related cellular vulnerability [14,15,16,17]. Retromer-dependent sorting and SORL1/SorLA-mediated trafficking further connect vesicular transport with APP metabolism and AD risk [18,19,20,21].

Large-scale human genetic studies have reinforced the view that AD susceptibility is distributed across pathways related to Aβ, tau, lipid metabolism, immunity, endocytosis and intracellular trafficking [22,23,24]. This convergence suggests that APP-associated biology should be examined not only at the level of APP cleavage products, but also through the protein environments in which endogenous APP is recovered. In parallel, APP and APP family proteins have been linked to synaptic vesicle biology, neurite structure, intercellular adhesion and synaptic transmission [25,26,27,28,29,30]. These observations raise the possibility that APP-associated protein contexts may connect vesicular trafficking with neuronal architecture and synaptic function.

Affinity purification coupled to mass spectrometry has provided important insights into protein-associated networks and has been applied to APP and neurodegeneration-related proteins [31,32,33,34]. APP nevertheless presents specific challenges for proteomic mapping because it is membrane-associated, proteolytically processed, and distributed across multiple dynamic compartments; recovered proteins can therefore depend on expression level, extraction conditions, antibody background, and bead binding [31,32,33,34,35]. Overexpression-based approaches may additionally alter APP stoichiometry, localization, and molecular proximity, making endogenous tagging an attractive strategy for preserving physiological expression [35,36,37]. Affinity-proteomic recovery can include direct binders, indirect complex members, compartmentally co-recovered proteins, and purification background, motivating evidence filtering and appropriate control comparison.

Here, we describe an endogenous *App* C-terminal 3×FLAG mouse resource and systematic cortical affinity proteomics under endogenous expression conditions. Accession-level recovery, quantitative evidence, peptide support, and background-aware GO analysis were integrated to define a cortical APP-associated candidate dataset for experimental follow-up.

## 2. Materials and Methods

### 2.1. Animals and Experimental Units

APP-3×FLAG knock-in mice on a C57BL/6J background and age-matched WT littermate controls were studied at eight weeks of age. APP-3×FLAG mice used for the biochemical, behavioral, and proteomic experiments were homozygous. The biochemical cohort comprised six males per genotype and the behavioral cohort seven males per genotype. For discovery affinity proteomics, cortical material from three APP-3×FLAG males was combined before affinity purification. Mice were housed under specific-pathogen-free conditions on a 12 h light/12 h dark cycle with food and water available ad libitum. All animal procedures were approved by the Institutional Animal Care and Use Committee (IACUC) of Fujian Provincial Hospital (approval code IACUC-FPH-SL-20260302[0198]; approval date: 2 March 2026).

### 2.2. Generation and Genotyping of the Tagged Allele

The mouse line was generated by Cyagen Biosciences using CRISPR/Cas9-mediated editing of the *App* locus, referenced to transcript NM_001198823.1. The gRNA-A1 target sequence was GCCGCTGCTGTGGCGCGGGTGGG. The donor introduced a 66 bp C-terminal 3×FLAG cassette (GACTACAAAGACCATGACGGTGATTATAAAGATCATGATATCGATTACAAGGATGACGATGACAAG), encoding DYKDHDGDYKDHDIDYKDDDDK, immediately upstream of the endogenous stop codon in exon 18. F0 founders were screened by junction PCR and sequence analysis and were crossed with WT C57BL/6J mice to establish F1 animals. F1 offspring were evaluated using two long-junction PCR assays. The 5′ junction assay used F1 (5′-TGAAGTCTGTCCTGAAGTCTGTCT-3′) and R1 (5′-CTTACTTGTCATCGTCATCCTTG-3′), with an expected targeted product of approximately 1.9 kb; the 3′ junction assay used F2 (5′-GCAGAACGACTACAAAGACCATG-3′) and R2 (5′-TCAGTGGTTTGTAACTCGCTTCCGT-3′), with an expected targeted product of approximately 2.5 kb. Sequencing primers were F3 (5′-GTGTCCTGTTACTGCCTACGAGTA-3′) and R3 (5′-GTAATGTAGAGACCAGAATGTA-3′). Routine zygosity genotyping used an allele-discriminating F5/R5 assay [F5, 5′-GGATCTCTGTACCTGCTTTCTA-3′; R5, 5′-TCAGGCATCTACTTGTGTTAC-3′], which yields a 358 bp targeted-allele product and a 292 bp WT product, together with a target-specific F4/R3 assay [F4, 5′-CAAGGATGACGATGACAAGTAAG-3′; R3 as above], which yields a 258 bp targeted product. Homozygous targeted mice show 358 and 258 bp products, heterozygotes show 358, 258, and 292 bp products, and WT mice show the 292 bp product. The APP-3×FLAG mice used for biochemical, behavioral, and proteomic experiments were homozygous, and WT mice were littermate controls. Primer and genotyping information is summarized in Table S1.

### 2.3. Brain Lysates and Immunoblotting

Mice were anesthetized by intraperitoneal injection of 100 µL of 10% (*w*/*v*) chloral hydrate before euthanasia and tissue collection. Brains were dissected on ice. For biochemical assays, whole-brain tissue was homogenized in 50 mM Tris-HCl, pH 7.4, 150 mM NaCl, 1% NP-40, 1 mM EDTA, and protease/phosphatase inhibitors. Lysates were clarified at 20,000× *g* for 15 min at 4 °C, and protein concentration was measured using the Pierce bicinchoninic acid (BCA) assay. Proteins were separated by SDS-PAGE and transferred to membranes for APP, FLAG, and β-actin detection. APP densitometry was normalized to β-actin.

### 2.4. Aβ Measurements

Amyloid-β (Aβ)40 and Aβ42 were measured in brain extracts from six WT and six APP-3×FLAG mice using an Aβ-specific immunoassay. WT and APP-3×FLAG samples were processed in parallel. Aβ40 and Aβ42 concentrations were expressed in pg/mg. For each animal, the Aβ42/Aβ40 ratio was calculated as r_i_ = Aβ42_i_/Aβ40_i_. The normalization constant C was defined as the mean of the individual r_i_ values in the WT group, and each WT and APP-3×FLAG value was expressed as y_i_ = r_i_/C.

### 2.5. Behavioral Assays

The behavioral cohort consisted of seven male mice per genotype and completed open-field, Y-maze spontaneous-alternation, and novel-object-recognition assays. Behavioral testing and behavioral outcome assessment were conducted under double-blind conditions, with investigators unaware of genotype allocation until completion of the assessments. Mice were housed under specific-pathogen-free conditions and were transferred to the behavioral testing room for a 30 min acclimatization period before testing. All experimental groups underwent the same acclimatization procedure. Recorded endpoints included total distance, mean velocity, center-area duration, spontaneous alternation, the training-phase Cognitive index, and the test-phase Recognition index. For novel-object recognition, the Cognitive index was calculated as 100 × T_a_/(T_a_ + T_b_), where T_a_ and T_b_ are exploration times for the two training objects, and the Recognition index was calculated as 100 × T_novel_/(T_novel_ + T_familiar_).

### 2.6. Computational Assessment of Tag Placement

To assess the structural context of the C-terminal tag, five APP770 and five APP770-3×FLAG predictions were generated using ColabFold/AlphaFold2 on 12 April 2025. The modeled fusion sequence appended DYKDHDGDYKDHDIDYKDDDDK to APP770. Quantitative analyses compared predicted local distance difference test (pLDDT) profiles, local Cα root-mean-square deviation (RMSD) in shared high-confidence APP segments, and pairwise ensemble RMSD. Contiguous segments of at least five residues with pLDDT > 70 in both model sets were aligned using the Kabsch method. pLDDT was interpreted as a per-residue measure of local model confidence. Low scores in the appended tag and adjacent C-terminal tail were treated as uncertainty in the predicted conformation, which may be compatible with flexibility but does not establish native disorder or a tag-induced structural perturbation [38].

### 2.7. Affinity Purification and Sample Input

For APP affinity enrichment, cortical lysates from three APP-3×FLAG males were prepared separately, and 20 mg of total protein from each mouse was combined, giving 60 mg total protein in 30 mL of lysate. The combined material was incubated overnight at 4 °C with 300 μL of anti-FLAG beads (Sigma, St. Louis, MO, USA) to enrich APP-3×FLAG and co-recovered proteins. The control purification used IgG with Protein A/G beads. Immunoprecipitation included five lysis-buffer washes followed by a final ammonium-bicarbonate wash before sample processing.

### 2.8. Protein Digestion and Peptide Preparation

Beads were washed with 1 mL of 50 mM ammonium bicarbonate, centrifuged at 1000 rpm for 2 min, and the supernatant was removed. Samples were reduced with 2 μL of 0.5 M tris(2-carboxyethyl)phosphine (TCEP) at 37 °C for 60 min and alkylated with 4 μL of 1 M iodoacetamide for 40 min at room temperature in the dark. Five volumes of acetone prechilled to −20 °C were added, and proteins were precipitated overnight at −20 °C. After centrifugation at 12,000× *g* for 20 min at 4 °C, the pellet was washed twice with 1 mL of prechilled 90% acetone, dried at room temperature, resuspended in 100 μL of 100 mM triethylammonium bicarbonate (TEAB), and digested overnight at 37 °C with approximately 2 μg of trypsin. Protein yield after FLAG enrichment was not quantified before digestion. Peptides were desalted using Pierce C18 Spin Tips, dried in a SpeedVac concentrator (Thermo Fisher, Waltham, MA, USA), and stored at −80 °C.

### 2.9. NanoLC and Tandem Mass Spectrometry

Peptides were analyzed on an Orbitrap Exploris 480 coupled to an EASY-nanoLC 1200 (Thermo Fisher, Waltham, MA, USA). The analytical column was a self-packed C18 column (15 cm × 75 μm i.d., 1.7 μm particle size), operated at 40 °C and 300 nL/min. Mobile phase A was 0.1% formic acid in water, and mobile phase B was 80% acetonitrile with 0.1% formic acid; the electrospray voltage was 2 kV. The sample injection volume was 3 μL, corresponding to approximately 1 μg of peptide. Positive-ion data-dependent acquisition used a 2 s cycle. MS1 covered *m*/*z* 350–1200 at 120,000 resolution with normalized automatic gain control (AGC) target 300% and a 100 ms maximum injection time. MS2 used 30,000 resolution, normalized AGC target 200%, a 50 ms maximum injection time, a 4 *m*/*z* isolation window, and stepped higher-energy collisional dissociation (HCD) at 25%, 30%, and 35%. Monoisotopic precursor selection was enabled with a minimum precursor intensity of 10,000 and charge states 2–6. Precursors were dynamically excluded for 30 s with a ±10 ppm tolerance. One microscan was specified at each level, with profile MS1 and centroid MS2.

### 2.10. Database Search and Exported Protein Evidence

Tandem mass spectra were analyzed using PEAKS Studio version 13 (Bioinformatics Solutions Inc., Waterloo, ON, Canada) against the UniProt Mus musculus 2025 database containing 20,773 searched entries. Trypsin was specified with semi-specific cleavage and up to two missed cleavages. Precursor- and fragment-ion tolerances were 10 ppm and 0.02 Da, respectively. Carbamidomethylation (+57.02 Da) was fixed; methionine oxidation (+15.99 Da), asparagine/glutamine deamidation (+0.98 Da), and protein N-terminal acetylation (+42.01 Da) were variable. FDR estimation used the PEAKS target-decoy workflow with an automatically generated decoy database. Peptide-sequence and protein-group identifications were filtered at 1% FDR, with at least one unique peptide required per reported protein group; resulting peptide-spectrum match (PSM)-level FDRs were 0.8% for APP and 0.4% for control. PEAKS PTM was enabled, and SPIDER was disabled. For downstream analysis, representative non-contaminant protein accessions were retained from the PEAKS protein output. De novo-only spectra were evaluated separately from the target-decoy-controlled database search. Because the standard mouse target database did not contain the engineered APP–3×FLAG fusion sequence, junction-spanning de novo assignments were compared directly with the engineered allele sequence and expected tryptic context. Junction-spanning assignments were kept separate from database-search FDR calculations and downstream candidate and GO analyses. A summary of the proteomics runs and LC-MS/MS acquisition settings is provided in Tables S2 and S3. Junction-spanning sequence evidence is provided in Table S4.

### 2.11. Protein Quantification and Missing-Value Handling

Protein-group identifiers and representative members were taken from the PEAKS Protein Group and Top fields, respectively; #Unique and #Spec provided the group-level unique-peptide and matched-spectrum counts. Protein Area was used as the condition-specific quantitative measure for APP-IP and control samples. APP-IP and control samples were searched independently without cross-run retention-time alignment, match-between-runs/identification transfer, or between-run normalization. Peptide-level Area represents an MS1 chromatographic feature area, and shared/non-unique peptides were excluded from protein-Area calculation. Missing protein Area values were not replaced by zero, imputed, or assigned a pseudocount. Protein identification was defined by presence in the PEAKS protein list, whereas quantitative availability required a positive Area value.

### 2.12. Candidate Filtering

The primary candidate filter required a positive APP-IP Area, PEAKS #Unique ≥2, not classified within the predefined background categories, and either no control identification or a raw APP/control Area ratio of at least four when positive Areas were available in both conditions. Shared identifications lacking a control Area were excluded from the final candidate set but were not treated as zero. APP (P12023) was treated as the bait and excluded from candidate counts. Background exclusions included immunoglobulin-related proteins, hemoglobins, keratins, albumin, and reverse/decoy entries. Sensitivity analyses varied the minimum unique-peptide requirement (one, two, or three) and the shared Area-ratio cutoff (two, four, or eight) across all nine combinations. The primary filter used at least two unique APP-IP peptides and a fourfold shared-Area cutoff. Accession-level evidence, retained candidates, background exclusions, and threshold-sensitivity results are provided in Tables S5–S8.

### 2.13. Gene Ontology Annotation and Overrepresentation

Mouse protein annotations were retrieved from UniProtKB release 2026_03 (2 September 2026) using organism ID 10090 [39]. Only exact accession matches were retained. Of 1874 source accessions, 1867 mapped exactly; the seven unmatched accessions were immunoglobulin entries excluded by the background filter. Direct GO biological-process (BP) and cellular-component (CC) memberships were parsed from the returned GO identifiers and names without ancestor propagation. The query was held fixed at the 990-candidate set and intersected with the annotated universe for each domain. Three nested backgrounds were evaluated: the observed union of proteins identified in either purification after exclusion of the APP bait and predefined background proteins; the subset with positive APP-IP Area; and the evidence-eligible subset additionally supported by at least two unique APP-IP peptides (n = 1003). The observed-union analysis used BP query/background sizes of 956/1730 and CC sizes of 972/1761. In the evidence-eligible analysis, the corresponding sizes were 956/969 and 972/985. Within every background and domain, all directly annotated terms with 10–500 background proteins were tested, including zero overlaps. One-sided hypergeometric upper-tail probabilities were calculated from complete memberships. Benjamini–Hochberg correction was applied separately to each complete background-by-domain family. The observed-union families contained 358 BP and 207 CC tests, whereas the evidence-eligible families contained 179 BP and 145 CC tests. Fold enrichment was calculated as (query overlap/query size)/(background term size/background size). For graphical presentation, ten terms per domain were selected according to observed-union BH-adjusted *p*-value and GO identifier, and the same terms were evaluated under the alternative background. Accession mapping and direct GO memberships are provided in Tables S9 and S10.

### 2.14. Statistical Analysis

Biochemical and behavioral analyses were performed in GraphPad Prism version 10.1.2 (GraphPad Software, San Diego, CA, USA). WT-versus-APP-3×FLAG comparisons used two-tailed unpaired Student’s *t*-tests and are presented as mean ± SEM. No separate normality test, formal homogeneity-of-variance test, or family-wise multiple-comparison adjustment was performed for these pairwise comparisons; *p*-values are therefore unadjusted pairwise results. Proteomic outputs from the pooled preparation were summarized descriptively. GO overrepresentation was evaluated using one-sided hypergeometric tests with Benjamini–Hochberg correction. Pairwise ensemble RMSD values were summarized descriptively across the five predictions generated for each sequence.

### 2.15. Targeted Biochemical Validation

To examine biochemical co-recovery of selected proteomic candidates, we performed targeted anti-FLAG co-immunoprecipitation followed by immunoblotting. ANXA2, DNM1, ATP6V1B2, RACK1, and UBE3A were selected from the retained candidate set on the basis of trafficking/proteostasis relevance and the availability of antibodies suitable for mouse immunoblotting. Cortical tissue from one APP-3×FLAG mouse was homogenized under non-denaturing conditions using Thermo Scientific Pierce IP Lysis Buffer (Cat. 87788). One twentieth of the lysate was retained as the input fraction, and 20 µg total protein was loaded for the input lane. The remaining lysate was divided between an IgG-control immunoprecipitation and anti-FLAG affinity capture, with 40 mg total protein assigned to each arm. The IgG-control arm received 40 µL IgG antibody, and the anti-FLAG arm received 40 µL anti-FLAG M2 Affinity Gel (Sigma-Aldrich, A2220). Samples were incubated for 3 h at 4 °C and washed four times. Bound proteins were resuspended in 1× loading buffer, heated at 95 °C for 10 min, separated by SDS-PAGE, and analyzed by immunoblotting.

All primary antibodies were used at 1:1000: ATP6V1B2 (Thermo Fisher Scientific, Waltham, MA, USA; Cat. No. PA5-52518), ANXA2 (Santa Cruz Biotechnology (Santa Cruz Biotechnology, Dallas, TX, USA; Cat. No. sc-47696), Dynamin I (Santa Cruz Biotechnology, Dallas, TX, USA; Cat. No. sc-12724), RACK1 (Santa Cruz Biotechnology, Dallas, TX, USA; Cat. No. sc-17754), UBE3A (Cell Signaling Technology, Danvers, MA, USA; Cat. No. 7526T), FLAG [rabbit anti-FLAG (DYKDDDDK) antibody; Shenzhen Kangti Biopharmaceutical Technology Co., Ltd., Shenzhen, China; Cat. No. KTSM1203], and APP [anti-amyloid precursor protein antibody (Y188); Abcam, Cambridge, UK]. Candidate signals were evaluated at the expected molecular-weight regions in relation to the input and IgG-control lanes. The targeted immunoblots were evaluated qualitatively.

## 3. Results

### 3.1. Generation and Verification of the Endogenous App C-Terminal 3×FLAG Allele

**Figure 1:**
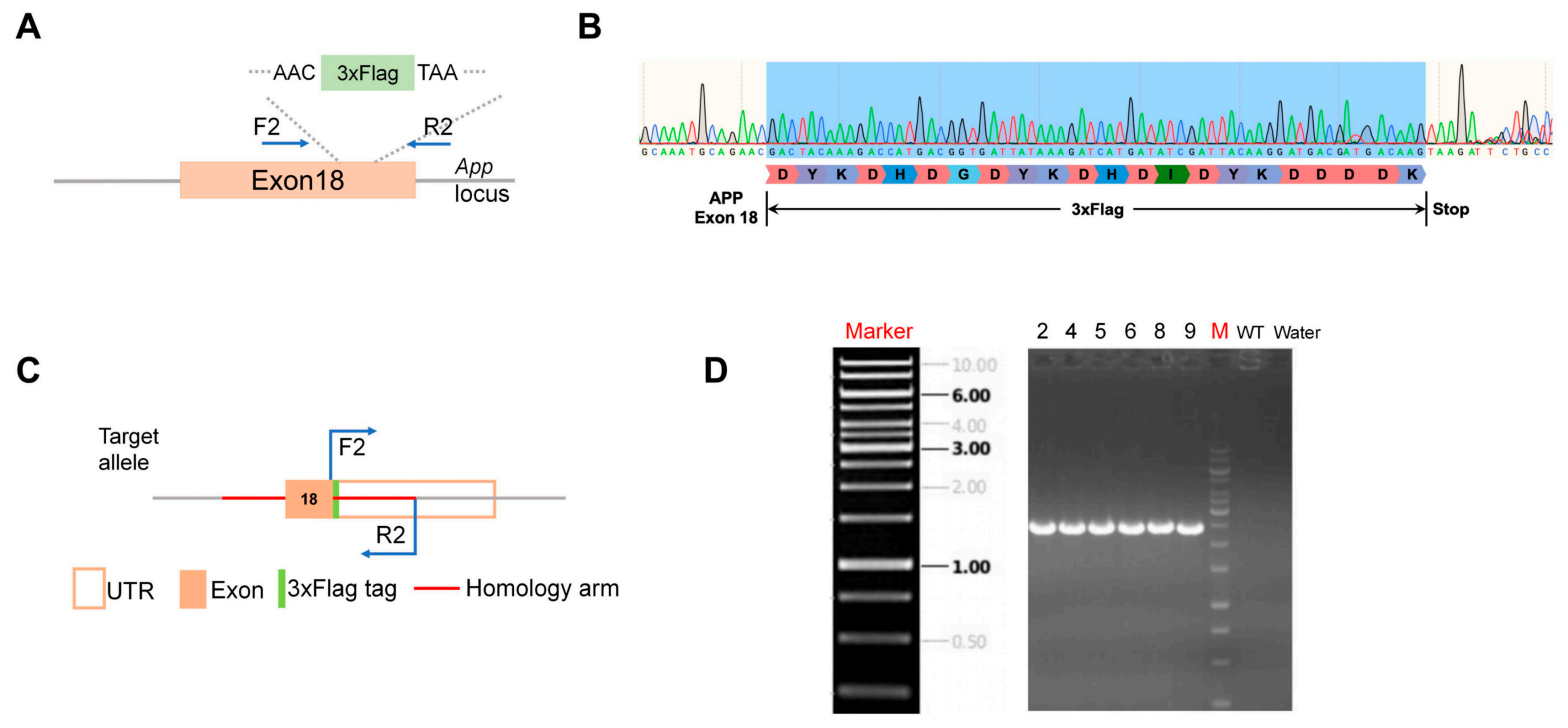
Generation of the endogenous *App* C-terminal 3×FLAG allele. (**A**) Targeting schematic showing placement of the 66 bp C-terminal 3×FLAG cassette immediately upstream of the exon 18 stop codon. (**B**) Representative Sanger junction chromatogram showing the in-frame tag sequence at the targeted allele. (**C**) Schematic of the targeted allele and F2/R2 3′-junction PCR configuration. (**D**) Representative PCR gel from F1 junction screening showing the targeted allele in animals 2, 4, 5, 6, 8, and 9. Primer information is provided in Table S1.

To recover APP-associated proteins from mouse brain under endogenous expression conditions, we established a C-terminally tagged *App* knock-in mouse line. A 66 bp cassette encoding the 22-residue 3×FLAG sequence DYKDHDGDYKDHDIDYKDDDDK was inserted at the end of *App* exon 18 immediately upstream of the endogenous stop codon, thereby positioning the affinity tag at the APP C-terminus while preserving the upstream APP coding sequence (Figure 1A). Sanger sequencing across the engineered region confirmed the expected in-frame *App*–3×FLAG junction and downstream TAA stop codon (Figure 1B), providing nucleotide-level confirmation of the intended C-terminal fusion. The 5′ and 3′ junction assays yielded the expected products of approximately 1.9 kb and 2.5 kb, respectively (Table S1), with the F2/R2 3′-junction configuration illustrated in Figure 1C. Routine zygosity genotyping distinguished the targeted and WT alleles using the F5/R5 and F4/R3 assays (Table S1). Consistent with the targeting design, the representative F1 junction-screening gel showed the expected targeted products in all six displayed animals (IDs 2, 4, 5, 6, 8, and 9) (Figure 1D). The APP-3×FLAG mice used for subsequent biochemical, behavioral, and proteomic analyses were homozygous, with WT littermates used as controls. Together, these data confirm the expected genomic configuration of the *App*–3×FLAG line.

### 3.2. Baseline APP and Aβ Measurements and Model-Based Assessment of the C-Terminal Tag

**Figure 2:**
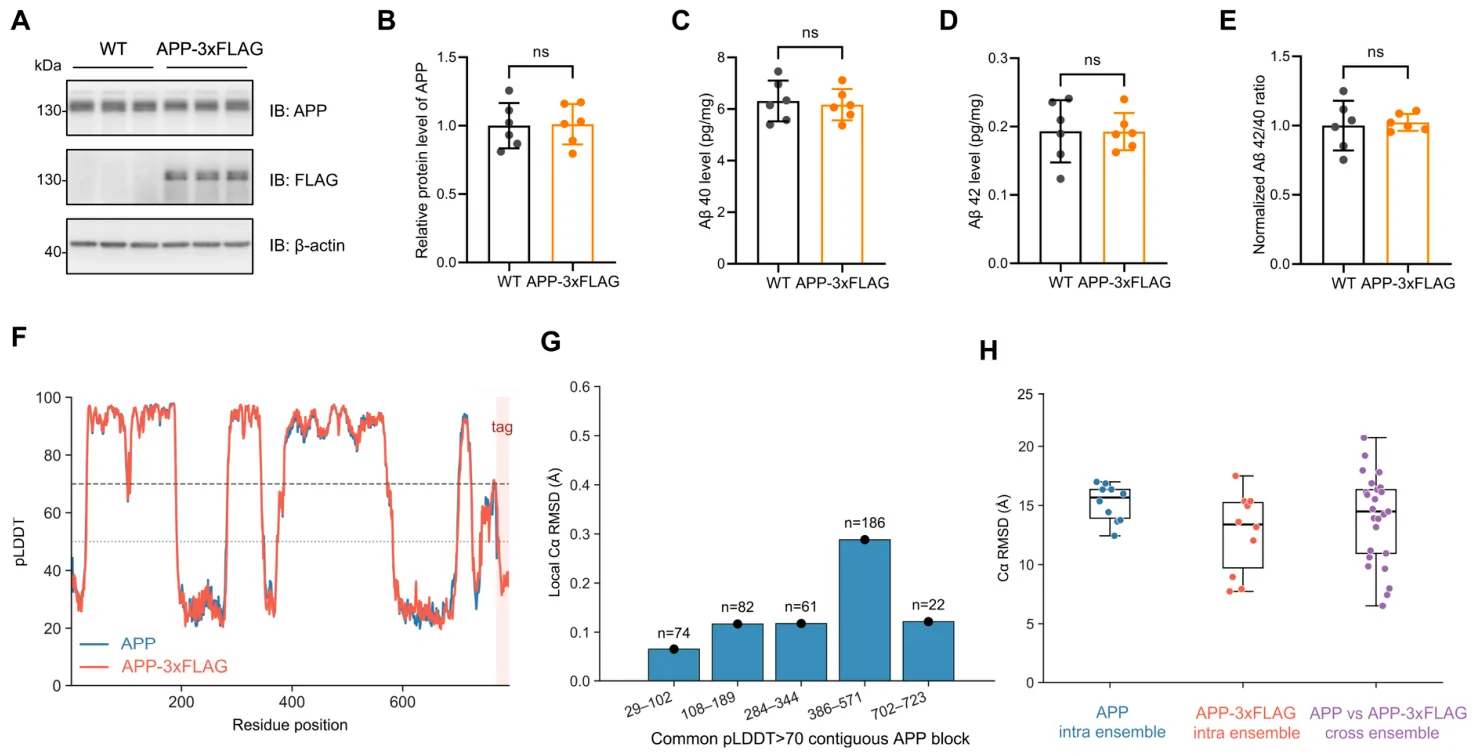
Baseline APP and Aβ measurements and model-based assessment of C-terminal 3×FLAG placement. (**A**) APP and FLAG immunoblots with β-actin loading control, showing three representative animals per genotype from the biochemical cohort. (**B**) APP abundance normalized to β-actin. (**C**) Aβ40 concentration. (**D**) Aβ42 concentration. (**E**) Normalized Aβ42/Aβ40 ratio. Each point represents one mouse (n = 6 per genotype); bars show mean ± SEM. Comparisons were performed using two-tailed unpaired Student’s *t*-tests. (**F**) ColabFold/AlphaFold2 pLDDT profiles for APP770 and APP770-3×FLAG predictions. pLDDT reports per-residue local model confidence; low scores in the appended 3×FLAG region and adjacent APP C-terminal tail indicate uncertain local conformations. (**G**) Local Cα RMSD after Kabsch alignment of five contiguous APP regions with pLDDT >70 in both model sets (residues 29–102, 108–189, 284–344, 386–571, and 702–723). These local alignments do not assess the appended tag or low-confidence C-terminal tail. (**H**) Pairwise ensemble RMSD distributions within APP predictions, within APP-3×FLAG predictions, and across APP versus APP-3×FLAG predictions. ns, not significant.

After establishing the targeted allele, we examined baseline APP abundance and Aβ measurements in WT and APP-3×FLAG mice. Immunoblotting detected APP in both WT and APP-3×FLAG brain lysates, whereas FLAG immunoreactivity was restricted to the tagged samples; β-actin served as the loading control (Figure 2A). APP abundance normalized to β-actin was comparable between genotypes, with no significant difference detected in six animals per group (Figure 2B). Aβ40 and Aβ42 concentrations likewise showed no significant WT-versus-APP-3×FLAG differences (Figure 2C,D), and the normalized Aβ42/Aβ40 endpoint remained comparable between genotypes (Figure 2E). Thus, C-terminal tagging was not accompanied by a detectable change in the measured steady-state APP abundance or Aβ readouts before proteomic profiling. We next compared APP770 and APP770-3×FLAG predictions to assess the modeled structural context of the C-terminal tag. The two model sets exhibited closely overlapping pLDDT profiles across the shared APP sequence, with low-confidence regions occurring in both; the appended 3×FLAG region and adjacent APP C-terminal tail also had low pLDDT scores (Figure 2F). These scores indicate limited confidence in the predicted local conformations. Quantitative local comparison was restricted to shared APP segments with pLDDT > 70 in both model sets. Five common high-confidence regions spanning residues 29–102, 108–189, 284–344, 386–571, and 702–723 all showed local Kabsch-aligned Cα RMSD values below 0.3 Å (Figure 2G), indicating close agreement of the predicted local backbone geometry across the confidently modeled APP regions. We next compared broader model-to-model variability across the prediction ensembles. The median APP-versus-APP-3×FLAG cross-ensemble RMSD was approximately 14.49 Å, whereas the largest intra-ensemble RMSD observed within either prediction set was approximately 17.49 Å (Figure 2H). The ensemble comparison was treated descriptively and does not establish structural equivalence of the two sequences. Representative APP770, APP770-3×FLAG, and overlay models are shown in Figure S1A–C. Together, these analyses support close agreement of the modeled high-confidence APP regions, while leaving the conformation of the appended tag and adjacent low-confidence tail unresolved.

### 3.3. Baseline Behavioral Measurements Show No Detectable Genotype-Associated Differences

**Figure 3:**
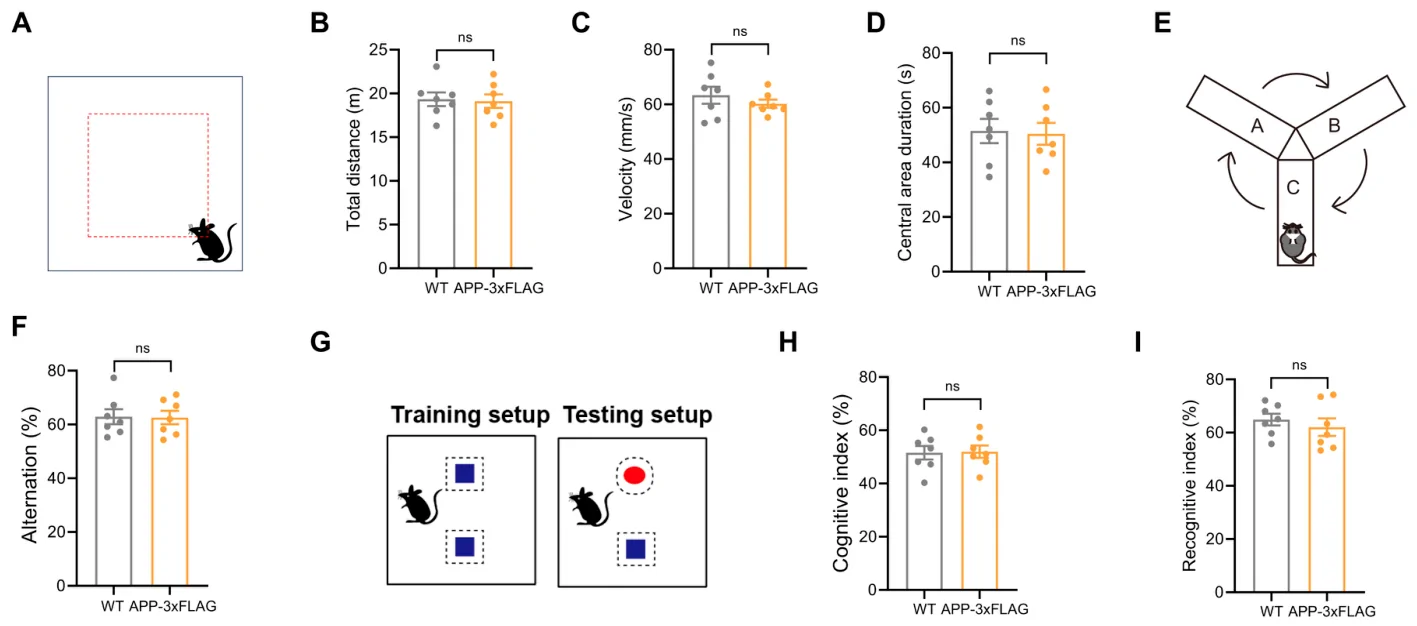
Baseline behavioral measurements. (**A**) Open-field schematic. (**B**) Total distance. (**C**) Mean velocity. (**D**) Central-area duration. (**E**) Y-maze schematic, with A, B, and C indicating the three arms of the maze. (**F**) Spontaneous alternation percentage. (**G**) Novel-object-recognition training and testing schematic. (**H**) Training-phase Cognitive index. (**I**) Test-phase Recognition index. Each point represents one mouse (n = 7 per genotype); bars show mean ± SEM. Comparisons were performed using two-tailed unpaired Student’s *t*-tests. ns, not significant.

We next assessed baseline behavior in seven male mice per genotype. The open-field paradigm is shown in Figure 3A. Total distance traveled (Figure 3B), mean velocity (Figure 3C), and central-area duration (Figure 3D) were comparable between WT and APP-3×FLAG mice, with no statistically significant genotype-associated differences detected across these locomotor and exploratory measures. Spatial working-memory-related performance was next assessed using the Y-maze paradigm shown in Figure 3E. Spontaneous alternation percentages were similar between genotypes, and the pairwise comparison did not reach statistical significance (Figure 3F). Novel-object recognition was then assessed during the training and test phases illustrated in Figure 3G. The training-phase Cognitive index showed no significant difference between WT and APP-3×FLAG mice (Figure 3H), and the test-phase Recognition index likewise did not differ significantly between genotypes (Figure 3I). Across all quantified behavioral endpoints, no significant genotype-associated differences were detected.

### 3.4. APP Affinity Proteomics Defines an Evidence-Filtered 990-Candidate Dataset

**Figure 4:**
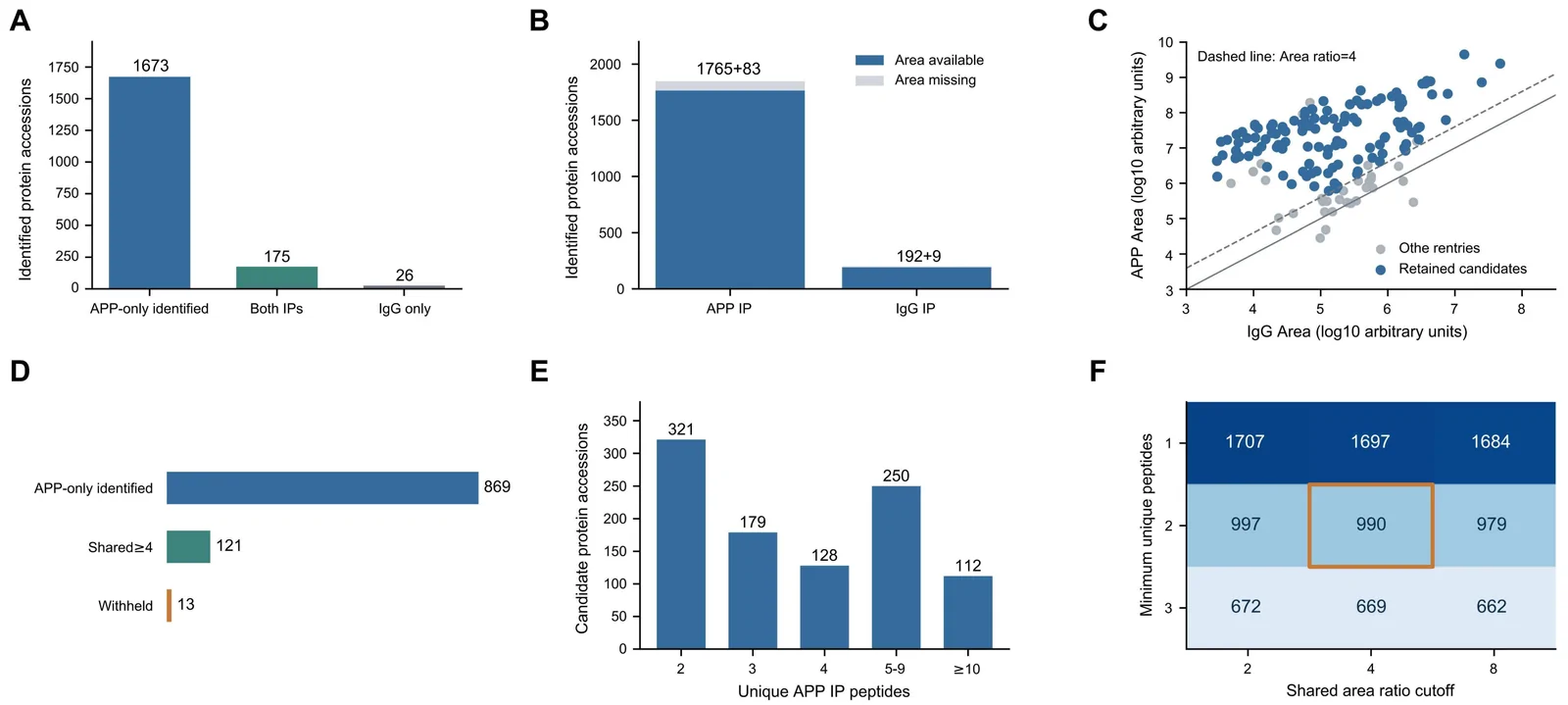
Recovery and evidence filtering. (**A**) Identification overlap among APP-IP-only, shared, and IgG-only accessions. (**B**) Availability of protein-level Area values in the APP-IP and control datasets. (**C**) APP versus IgG Area for shared quantified accessions; the dashed line marks an APP/IgG Area ratio of 4. (**D**) Candidate retention after control comparison among 1003 evidence-eligible non-bait accessions: 869 APP-IP-only and 121 shared quantified accessions with Area ratio ≥4 were retained, whereas 13 were not retained. (**E**) Distribution of unique APP-IP peptide support among the 990 retained candidates. (**F**) Sensitivity of retained-candidate counts to the minimum unique-peptide requirement and shared Area-ratio cutoff; the orange frame highlights the primary filtering criterion of at least two unique APP-IP peptides and a fourfold shared Area-ratio cutoff, yielding 990 retained candidates.

We next used cortical anti-FLAG affinity purification followed by LC-MS/MS to characterize proteins recovered with endogenous APP-3×FLAG. The identification-overlap analysis separated APP-IP-only, shared, and IgG-control-only accessions (Figure 4A). The APP-IP and control datasets contained 1848 and 201 identified accessions, corresponding to 1681 and 179 protein groups, respectively. Merging the two datasets yielded 1874 distinct accessions, including 1673 APP-IP-only, 175 shared, and 26 control-only entries (Table S5). Protein-level Areas were available for 1765 APP-IP and 192 control accessions (Figure 4B). Among the 175 shared identifications, 166 had positive Areas in both runs, six lacked a control Area, and three lacked an APP Area. For the shared accessions quantified in both runs, APP-IP Area was plotted against control Area, with an APP/control Area-ratio threshold of four used as the reference for candidate filtering (Figure 4C). We next applied the control-comparison criterion to the evidence-eligible APP-IP set (Figure 4D). Before control comparison, 1003 accessions remained after exclusion of the bait and predefined background proteins and met the requirements of a positive APP-IP Area and at least two protein-group-specific unique APP-IP peptides, representing 979 APP-IP protein groups. The final filtering strategy retained 990 candidate accessions representing 967 APP-IP protein groups, including 869 APP-IP-only identifications and 121 shared quantified accessions with an APP/control Area ratio of at least four. Thirteen otherwise eligible accessions were not retained, including ten with ratios below four and three with a control identification but no quantitative control Area. APP itself was recovered with 19 unique peptides and 96 spectra and was excluded from candidate counts as the bait. Across the 990 retained candidates, the median number of unique APP-IP peptides was 3 (IQR, 2–6), the median matched APP-IP spectral count was 5 (IQR, 3–11), and the median APP-IP Area was 2.17 × 10^6^ (IQR, 7.83 × 10^5^–7.68 × 10^6^) (Figure 4E; Table S6). Accession-level evidence and filtering outcomes are provided in Tables S5–S7. Sensitivity analysis examined how candidate counts varied across related filtering thresholds (Figure 4F). At the two-peptide criterion, APP/control Area-ratio cutoffs of 2, 4, and 8 retained 997, 990, and 979 candidates, respectively, whereas increasing the requirement to at least three unique peptides at the fourfold cutoff retained 669 candidates (Table S8). Two de novo-only MS/MS assignments supported the engineered APP–3×FLAG junction: FFEQMQNDYK and its methionine-oxidized form FFEQM(+15.99)QNDYK (Table S4). Both assignments span the canonical APP C-terminus and the initial DYK residues of the FLAG cassette, providing peptide-level support for expression of the engineered fusion. Together, these analyses define a 990-entry APP affinity-proteomic candidate dataset integrating identification evidence, quantitative recovery, peptide support, control comparison, and threshold sensitivity. The resulting dataset provides a basis for biological interpretation and candidate selection for mechanistic follow-up.

### 3.5. Gene Ontology Analysis Identifies a Cellular-Component Signature in the APP-Associated Candidate Dataset

**Figure 5:**
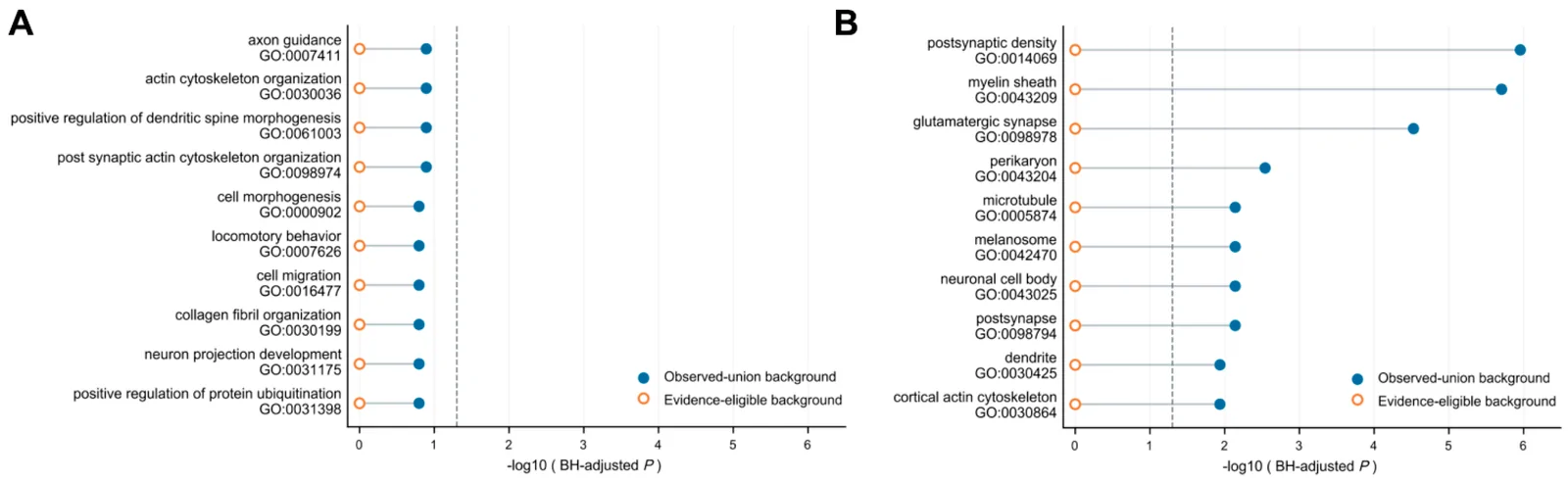
Gene Ontology analysis of the APP-associated candidate dataset. (**A**) Biological-process terms selected from the observed-union analysis and displayed under both the observed-union and evidence-eligible backgrounds. The BP terms with the lowest nominal *p*-values included axon guidance and actin cytoskeleton organization, although no BP term remained significant after Benjamini–Hochberg correction. (**B**) Cellular-component analysis identified 21 terms significant after Benjamini–Hochberg correction under the observed-union background, including postsynaptic density, myelin sheath, and glutamatergic synapse. For each term, values are shown for the observed-union and evidence-eligible backgrounds. Points indicate −log10 (Benjamini–Hochberg-adjusted *p*), and the dashed line denotes adjusted *p* = 0.05.

To characterize the functional organization of the 990-candidate dataset, we performed Gene Ontology overrepresentation analysis using experimentally defined protein backgrounds. In the biological-process analysis (Figure 5A), the observed-union background included 956 BP-annotated candidate accessions among 1730 annotated proteins across 358 eligible terms. The lowest nominal *p*-values under the observed-union background were observed for axon guidance (27/32; fold enrichment 1.53; raw *p* = 4.57 × 10^−4^) and actin cytoskeleton organization (42/55; fold enrichment 1.38; raw *p* = 8.35 × 10^−4^), although neither remained significant after Benjamini–Hochberg correction (minimum adjusted *p* = 0.128; Table S11). The corresponding observed-union universe summary is provided in Table S12. No BP term reached the adjusted significance threshold under the evidence-eligible background either (Table S13). In contrast, 21 of 207 eligible cellular-component terms remained significant after Benjamini–Hochberg correction under the observed-union background (Figure 5B). Among the most significant terms were postsynaptic density (113/146; fold enrichment 1.40; adjusted *p* = 1.11 × 10^−6^), myelin sheath (94/119; fold enrichment 1.43; adjusted *p* = 1.97 × 10^−6^), and glutamatergic synapse (183/265; fold enrichment 1.25; adjusted *p* = 2.97 × 10^−5^). Additional enriched terms included perikaryon, neuronal cell body, postsynapse, dendrite, microtubule, and cortical actin cytoskeleton (Table S11). This cellular-component pattern was preserved when the background was restricted to proteins with positive APP Areas, which again yielded 21 significant CC terms and no significant BP terms (Table S13). By contrast, further restriction to the evidence-eligible background substantially reduced the available contrast because the 990-candidate query occupied nearly the entire eligible universe. Under this background, no CC term remained significant. Consistent with this loss of contrast, postsynaptic density shifted from 113 of 146 background members to 113 of 113, myelin sheath from 94/119 to 94/96, and glutamatergic synapse from 183/265 to 183/184, bringing their fold-enrichment estimates close to unity. Protein-group-level analysis reproduced the same overall pattern. Using all APP-IP protein groups as the reference, 11 of 197 CC terms were significant, whereas no BP term was significant across 348 tests. Restricting the background to evidence-eligible APP groups yielded no significant BP or CC terms (Tables S14 and S15). Together, these analyses identify a cellular-component signature centered on postsynaptic, glutamatergic, myelin-associated, neuronal, and cytoskeletal compartments within the APP-associated candidate dataset. The accession- and protein-group-level analyses further show that enrichment strength depends on the experimentally defined reference background.

### 3.6. Biochemical Validation Supports ANXA2 and RACK1 Co-Recovery

To test selected proteomic candidates by an orthogonal biochemical readout, we performed anti-FLAG co-immunoprecipitation followed by immunoblotting of cortical lysate under non-denaturing conditions. Tagged APP was recovered in the anti-FLAG fraction, as confirmed by both FLAG and APP immunoblotting, with no comparable APP signal in the IgG-control lane (Figure S2A). ANXA2 was detected in the input and anti-FLAG fractions at the expected molecular-weight region, whereas the IgG-control lane showed no comparable band at that position. The DNM1 blot did not resolve a clear candidate-specific co-recovery signal under these conditions. For ATP6V1B2, overlap of the expected molecular-weight region with immunoglobulin heavy-chain signal prevented an unambiguous assignment (Figure S2B). RACK1 likewise showed a band in the input and anti-FLAG fractions without a comparable band at the corresponding position in the IgG-control lane. The UBE3A blot did not permit an unambiguous assignment of APP-associated co-recovery under the conditions tested (Figure S2C).

ANXA2 and RACK1 were APP-IP-only retained candidates in the discovery dataset, supported by 7 unique peptides/8 spectra and 4 unique peptides/7 spectra, respectively (Tables S6 and S16). Their biochemical co-recovery with APP-3×FLAG provides convergent support from mass spectrometry and targeted immunoblotting, identifying experimentally supported candidates for further investigation of APP-associated trafficking and proteostasis.

## 4. Discussion

By introducing a C-terminal 3×FLAG cassette into the endogenous App locus, we developed a mouse model that enables affinity recovery of APP-associated proteins without transgenic APP overexpression and used this system to generate a cortical affinity-proteomic dataset. Evidence-based filtering retained 990 candidate accessions for downstream mechanistic investigation. Junction PCR and Sanger sequencing verified the engineered App allele, while two distinct de novo-only MS/MS assignments converged on the expected APP–3×FLAG junction peptide. Baseline APP abundance, Aβ40, Aβ42, and normalized Aβ42/Aβ40 measurements showed no significant genotype-associated differences between WT and APP-3×FLAG mice. Computational modeling provided complementary structural context, with closely overlapping pLDDT profiles and local Cα RMSD values below 0.3 Å across five shared high-confidence APP regions. These findings support expression of the intended tagged allele, with no statistically significant genotype-associated differences detected in the baseline readouts examined and close agreement in the confidently modeled APP regions.

Previous studies of APP-associated proteins in the brain have used complementary experimental designs. Bai et al. applied time-controlled transcardiac cross-linking to examine APP-family protein proximity in intact brain and subsequently validated selected associations [34]. Kohli et al. purified tagged APP from transgenic mouse brain, compared protein recovery with WT controls using quantitative mass spectrometry, and performed orthogonal validation of selected candidates, including synaptotagmin-1 [35]. The present work differs principally in the use of an affinity tag introduced directly into the endogenous App locus, allowing APP-associated material to be recovered under endogenous expression control. This feature provides a complementary experimental framework for studying APP-associated protein recovery while avoiding the expression-level perturbations inherent to transgenic overexpression systems.

The targeted biochemical findings extend the proteomic resource by supporting ANXA2 and RACK1 association with APP-containing material recovered under endogenous expression conditions (Figure S2). ANXA2 has been linked to phosphorylated presenilin-1 and autophagosome–lysosome fusion, with consequences for APP-βCTF/Aβ metabolism, whereas RACK1 has been implicated in APP processing and translational quality control [40,41,42]. The present co-recovery results connect these biologically relevant candidates to APP-associated protein assemblies in mouse cortex and provide a basis for testing their contributions to APP trafficking and proteostasis.

The unresolved DNM1 and UBE3A immunoblot signals leave their potential association with APP open for further examination. Candidate-specific assessment could include alternative lysis-buffer compositions, detergent and salt concentrations, washing conditions, and antibodies recognizing different epitopes. These are possible methodological contributors to detection, not mechanisms established by the present data. ATP6V1B2 similarly requires improved discrimination from immunoglobulin heavy-chain signal. Such refinements could clarify the recovery of these candidates while extending the biochemical evidence established here for ANXA2 and RACK1.

The recovery of established APP-associated proteins in the same dataset provides an additional reference point for this interpretation. APBB2/Fe65L1 has direct structural evidence for binding the APP cytoplasmic tail; AP2A1 has been shown to associate with APP C-terminal fragments through a C-terminal AP2-recognition motif; CLSTN1 is co-transported with APP in neuronal axons and participates in APP-containing trafficking carriers; and SYT1 was previously validated as a direct APP-binding synaptic-vesicle protein [35,43,44,45]. Their presence among the retained candidates, together with the new ANXA2 and RACK1 co-recovery results, supports the utility of the endogenous APP-3×FLAG system for recovering both previously established and newly supported APP-associated proteins.

The biological composition of the recovered candidate set is consistent with the broad cellular itinerary of APP. APP moves through biosynthetic, secretory, plasma-membrane, endocytic, and endolysosomal compartments, and this trafficking determines access to secretases and pathways involved in fragment turnover [10,11,12,13]. Endosomal abnormalities reported in Alzheimer’s disease and Down syndrome, together with retromer- and SORL1-dependent regulation of APP metabolism, provide additional context for the recovery of trafficking-associated proteins [14,15,16,17,18,19,20,21,46]. Likewise, established roles of APP-family proteins in synaptic function, vesicular trafficking, and cell adhesion provide a framework for interpreting the postsynaptic, glutamatergic, neuronal, myelin-associated, and cytoskeletal annotations identified in the GO analysis [25,26,29]. These functional categories therefore provide a rational basis for prioritizing subsets of synaptic, trafficking, myelin-associated, and cytoskeletal candidates for targeted experimental follow-up.

Because the 3×FLAG cassette is fused to the APP C-terminus, proteolytic products retaining this terminus, including C99, C83, and the APP intracellular domain (AICD), also retain the tag and can potentially contribute to anti-FLAG recovery (Figure S3) [7,10,11]. Consequently, the candidate dataset may include proteins associated with full-length APP, C-terminal fragments, or both. The present affinity-proteomic and co-immunoprecipitation assays do not distinguish these sources or quantify the contribution of each APP-derived species. The ANXA2 and RACK1 findings therefore support association with FLAG-containing APP-derived assemblies without identifying the APP species involved. This distinction defines the molecular scope of the resource and motivates future full-length- and fragment-selective analyses.

The enrichment analyses further illustrate the importance of defining an appropriate reference universe in affinity-proteomic studies. Cellular-component overrepresentation was significant when the observed accession union was used as the background and remained evident when the background was restricted to proteins with positive APP Areas. In contrast, when the reference universe was narrowed to proteins already satisfying the APP peptide-evidence requirement, the 990-candidate query comprised most of the eligible background and the enrichment contrast largely disappeared. These results indicate that functional overrepresentation is not an intrinsic property of the candidate list alone but depends on the experimentally defined population against which that list is evaluated. Reporting this background sensitivity provides a more transparent interpretation of the GO results and limits overstatement of functional enrichment.

The discovery dataset was generated from a pooled cortical preparation with an IgG/Protein A/G purification control. This design supports descriptive candidate discovery; between-animal reproducibility remains to be established. Separately prepared APP-3×FLAG and WT cortices processed with the same anti-FLAG M2 resin would further assess reproducibility and resin-dependent background. The targeted ANXA2 and RACK1 findings add candidate-specific biochemical evidence to this resource. Reciprocal and species-resolved biochemical approaches could further define the composition of the recovered APP-associated assemblies.

The experiments were restricted to eight-week-old male mice and did not evaluate female animals. The resulting candidate dataset and the ANXA2/RACK1 co-recovery findings therefore describe the male cortical context examined here. Sex-specific differences in the composition or recovery of APP-associated assemblies cannot be assessed from these data, and the findings should not be assumed to apply unchanged to females. Comparative studies including both sexes will be needed to identify shared and sex-dependent associations. Extension to older ages and disease-relevant backgrounds would further define the biological scope of this resource.

The structural comparisons also have a defined scope. pLDDT measures confidence in the predicted local structure rather than biochemical stability or function [38]. The low scores in the appended 3×FLAG region and adjacent APP C-terminal tail indicate uncertain local conformations (Figure 2F). They may reflect conformational flexibility or limited predictive information; the present modeling does not distinguish these possibilities or establish native disorder. Low pLDDT alone is not evidence that the tag destabilizes APP. Conversely, close agreement within the five shared high-confidence APP segments does not establish preservation of the low-confidence tail or exclude tag-dependent effects on C-terminal associations (Figure 2G). The ensemble RMSD comparison is descriptive and does not resolve this limitation (Figure 2H). Thus, the modeling provides local structural context for the tagged resource rather than proof of functional neutrality. Complementary structural and interaction analyses would be needed to assess the C-terminal region in its native molecular environment.

Taken together, the study combines endogenous APP tagging, allele verification, baseline phenotyping, structural-model comparisons, accession-level proteomic evidence, and background-aware functional analysis with targeted biochemical support for ANXA2 and RACK1. This integrated resource enables the selection of experimentally grounded candidates for studies of APP-associated trafficking and proteostasis.

## 5. Conclusions

Endogenous C-terminal tagging of APP with 3×FLAG provides a genetically defined platform for recovering APP-associated proteins from mouse cortex under endogenous expression conditions. The resulting dataset integrates genomic validation of the engineered allele with baseline biochemical, Aβ, behavioral, structural-modeling, peptide-level, and quantitative proteomic evidence. Application of explicit evidence criteria retained 990 candidate accessions representing 967 APP-IP protein groups, while two de novo-only spectra supported recovery of the expected APP–3×FLAG junction peptide.

Functional annotation identified cellular-component enrichment under experimentally observed backgrounds and demonstrated its sensitivity to reference-universe definition. Targeted anti-FLAG co-immunoprecipitation and immunoblotting added biochemical support for ANXA2 and RACK1 co-recovery with APP-3×FLAG. Together, these findings connect proteomic nomination to experimental follow-up and establish a resource for investigating selected APP-associated protein assemblies, their molecular organization, and their roles in trafficking and proteostasis.

## Data Availability

The original mass-spectrometry proteomics data presented in this study are available through ProteomeXchange via the iProX partner repository under PXD081950 (parent project IPX0018716000; subproject IPX0018716001). During peer review, anonymous access is available at https://www.iprox.cn/page/SSV024.html;url=17854244574655XT0, accessed on 5 October 2026 (password: YNr5). Analysis code is available through the OSF view-only repository https://osf.io/xvgkf/?view_only=6033e65443d942049b3152042ad67ebc, accessed on 5 October 2026. Accession-level proteomic evidence, candidate-filter sensitivity analyses, GO mappings and test families, protein-group mappings, LC-MS/MS acquisition settings, genotyping information, and de novo APP–3×FLAG junction evidence are included in the Supplementary Materials. The APP-3×FLAG mouse line is available from the corresponding author on request, subject to institutional approvals and an applicable material transfer agreement. Further inquiries may be directed to the corresponding author.

## Supplementary Materials

The following supporting information can be downloaded at: https://www.mdpi.com/article/10.3390/biomedicines14102293/s1. Figure S1: Representative predicted model renderings of APP770 and APP770-3×FLAG; Figure S2: Targeted biochemical validation of selected APP-associated candidates; Figure S3: C-terminal tag retention by APP proteolytic species; Table S1: Genotyping and sequencing primers; Table S2: Proteomics run summary; Table S3: LC-MS/MS acquisition settings and gradient; Table S4: APP-3×FLAG de novo junction evidence; Table S5: Accession-level proteomic evidence; Table S6: Evidence-filtered candidates; Table S7: Background exclusion list; Table S8: Candidate-filter sensitivity; Table S9: UniProt accession mapping; Table S10: Direct GO memberships; Table S11: Observed-union GO overrepresentation; Table S12: Observed-union GO universe summary; Table S13: GO background-sensitivity analyses (Table S13A–D); Table S14: Accession-to-protein-group mapping; Table S15: Protein-group GO sensitivity analyses (Table S15A–E); Table S16: Targeted biochemical-validation summary.
