# Supplementary material for: Endogenous APP-Associated Proteomics in Mouse Brain Defines a Candidate Resource for APP Trafficking and Proteostasis Studies
Source: Biomedicines. 2026 Oct 9;14(10):2293. doi: 10.3390/biomedicines14102293 (PMC13701415; doi:10.3390/biomedicines14102293)
Supplement: Supplementary file 1 [file biomedicines-14-02293-s001.zip › Supplementary_Figures S1-S3.pdf]

Supplementary Figure

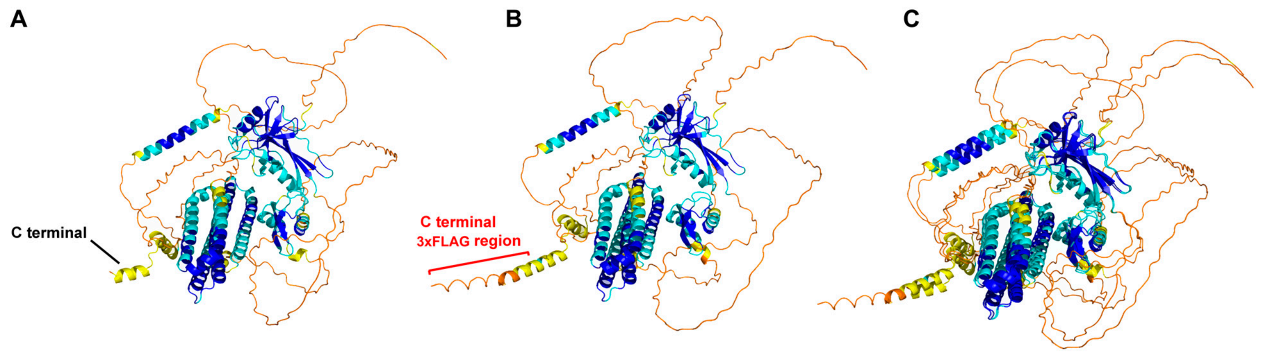

Figure S1 Representative predicted model renderings of APP770 and APP770-3×FLAG

(A) Representative ColabFold/AlphaFold2-predicted APP770 model with the C terminus indicated. (B) Representative APP770-3×FLAG predicted model with the appended C-terminal 3×FLAG region indicated. (C) Overlay of representative APP770 and APP770-3×FLAG predicted models.

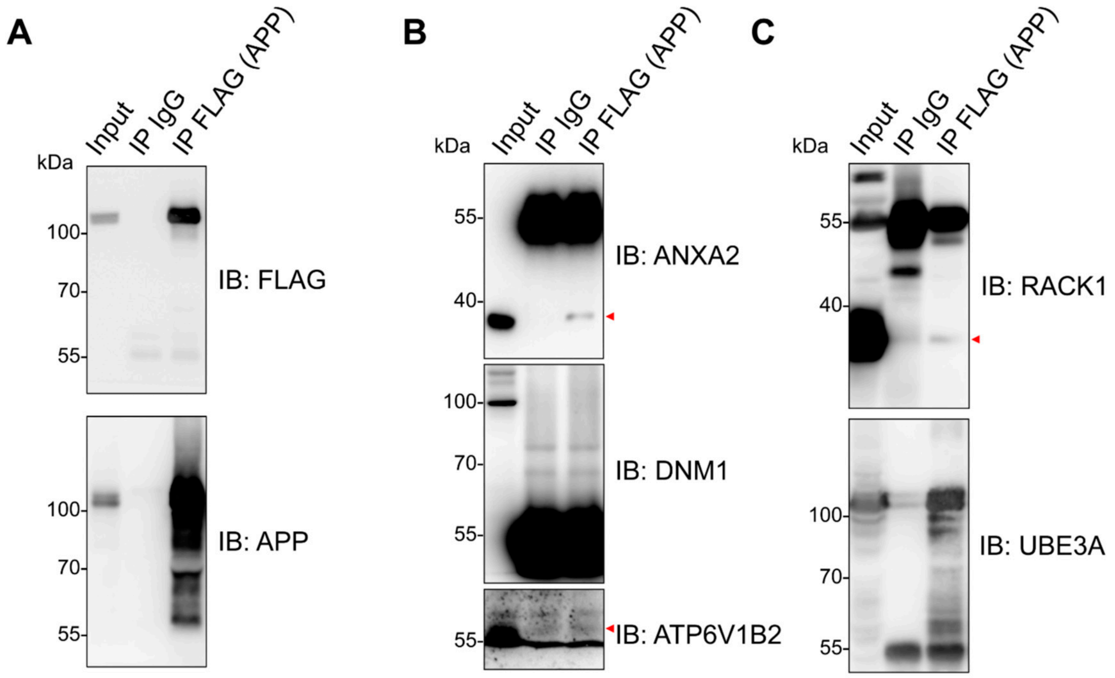

Figure S2. Targeted biochemical validation of selected APP-associated candidates.

APP-3×FLAG cortical lysate was subjected to IgG-control immunoprecipitation or anti-FLAG M2 affinity capture. (A) FLAG and APP immunoblotting confirmed recovery of tagged APP in the anti-FLAG fraction. (B) ANXA2 was detected in the input and anti-FLAG fractions without a comparable signal at the corresponding molecular-weight position in the IgG-control lane. DNM1 did not yield an unambiguous co-recovery signal under the conditions tested. ATP6V1B2 remained inconclusive because its expected molecular-weight region overlapped the immunoglobulin heavy-chain signal. (C) RACK1 was detected in the input and anti-FLAG fractions without a comparable signal at the corresponding position in the IgG-control lane. UBE3A did not yield an unambiguous co-recovery signal under the conditions tested. Red arrowheads indicate the expected molecular-weight regions of the indicated candidates. Biological n = 1; the same cortical preparation supplied the input and both IP arms.

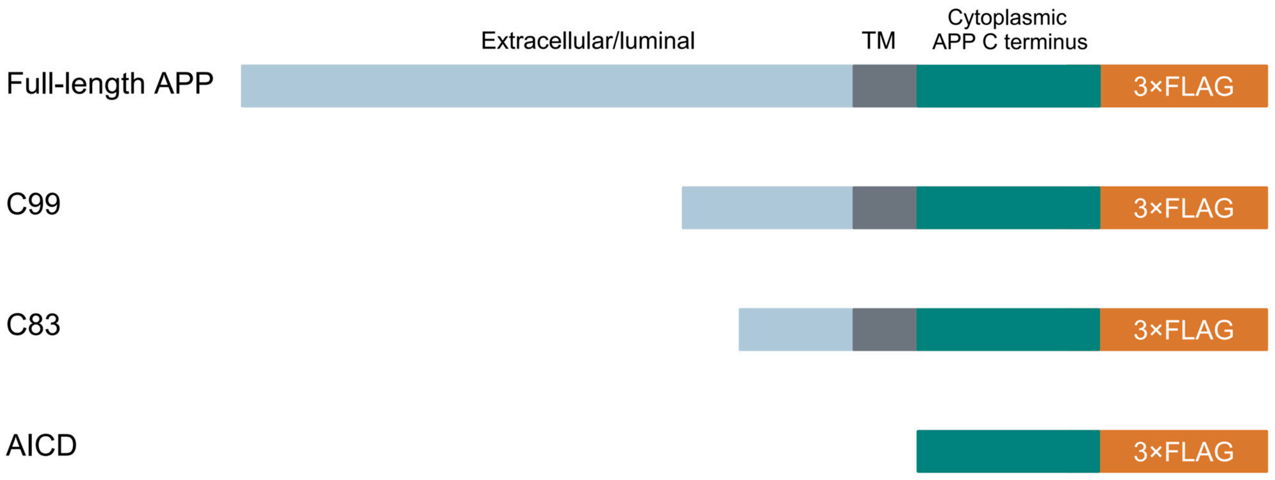

Figure S3. C-terminal tag retention by APP proteolytic species

Schematic illustrating retention of the C-terminal 3×FLAG tag by full-length APP, C99, C83, and AICD, and the resulting capacity of C-terminal affinity capture to sample multiple APP-derived molecular species.
